# Supplementary material for: A Humanized Yeast Phenomic Model of Deoxycytidine Kinase to Predict Genetic Buffering of Nucleoside Analog Cytotoxicity
Source: Genes (Basel). 2019 Sep 30;10(10):770. doi: 10.3390/genes10100770 (PMC6826991; doi:10.3390/genes10100770)
Supplement: Supplementary file 1 [file genes-10-00770-s001.zip › Additional_Files/Additional_File7_GOTermHeatmaps.zip!/Additional_File7_GOTermHeatmaps/spliceosomal_complex_assembly.pdf]

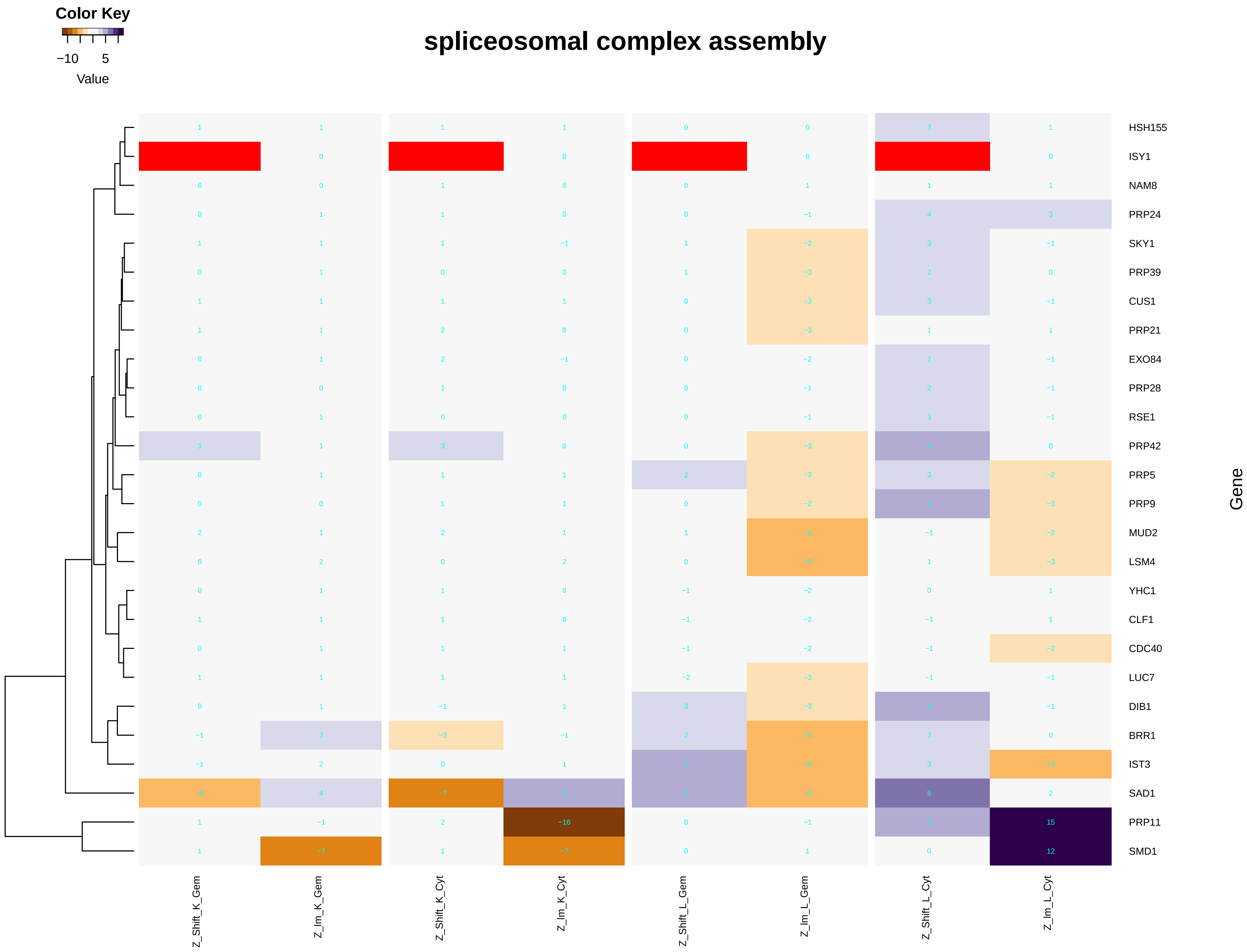
Color Key
−10
5
Value
spliceosomal complex assembly
HSH155
ISY1
NAM8
PRP24
SKY1
PRP39
CUS1
PRP21
EXO84
PRP28
RSE1
PRP42
PRP5
PRP9
MUD2
LSM4
YHC1
CLF1
CDC40
LUC7
DIB1
BRR1
IST3
SAD1
PRP11
SMD1
Gene
Z_Shift_K_Gem
Z_Im_K_Gem
Z_Shift_K_Cyt
Z_Im_K_Cyt
Z_Shift_L_Gem
Z_Im_L_Gem
Z_Shift_L_Cyt
Z_Im_L_Cyt
